# Silencing *Ditylenchus destructor* cathepsin L-like cysteine protease has negative pleiotropic effect on nematode ontogenesis

**DOI:** 10.1038/s41598-024-60018-5

**Published:** 2024-05-01

**Authors:** Guoqiang Huang, Ziwen Cong, Zhonglin Liu, Feng Chen, Alejandra Bravo, Mario Soberón, Jinshui Zheng, Donghai Peng, Ming Sun

**Affiliations:** 1 National Key Laboratory of Agricultural Microbiology, Huazhong Agricultural University, Wuhan, 430070 China; 2 Hubei Hongshan Laboratory, Wuhan, 430070 China; 3 Instituto de Biotecnología, Universidad Nacional Autónoma de México, 62210 Cuernavaca, Morelos Mexico

**Keywords:** Parasitology, Developmental biology

## Abstract

*Ditylenchus destructor* is a migratory plant-parasitic nematode that severely harms many agriculturally important crops. The control of this pest is difficult, thus efficient strategies for its management in agricultural production are urgently required. Cathepsin L-like cysteine protease (CPL) is one important protease that has been shown to participate in various physiological and pathological processes. Here we decided to characterize the CPL gene (*Dd-cpl-1*) from *D. destructor*. Analysis of *Dd-cpl-1* gene showed that *Dd-cpl-1* gene contains a signal peptide, an I29 inhibitor domain with ERFNIN and GNFD motifs, and a peptidase C1 domain with four conserved active residues, showing evolutionary conservation with other nematode CPLs. RT-qPCR revealed that *Dd-cpl-1* gene displayed high expression in third-stage juveniles (J3s) and female adults. In situ hybridization analysis demonstrated that *Dd-cpl-1* was expressed in the digestive system and reproductive organs. Silencing *Dd-cpl-1* in 1-cell stage eggs of *D. destructor* by RNAi resulted in a severely delay in development or even in abortive morphogenesis during embryogenesis. The RNAi-mediated silencing of *Dd-cpl-1* in J2s and J3s resulted in a developmental arrest phenotype in J3 stage. In addition, silencing *Dd-cpl-1* gene expression in female adults led to a 57.43% decrease in egg production. Finally, *Dd-cpl-1* RNAi-treated nematodes showed a significant reduction in host colonization and infection. Overall, our results indicate that Dd-CPL-1 plays multiple roles in *D. destructor* ontogenesis and could serve as a new potential target for controlling *D. destructor*.

## Introduction

The potato rot nematode *Ditylenchus destructor* is a migratory endoparasite of plants and is an important worldwide quarantine pest^1^. *D. destructor* can feed on a wide range of crops and even fungi, with the most devastating effects on potato, sweet potato and peanut, causing significant damage to global crop production^2^. It has been estimated that *D. destructor* induced an average loss of 10% in potato yield in America^3^, and 20% to 50% loss in sweet potato yield, that sometimes could increase up to 100% in China^4^. Although *D. destructor* causes heavy losses to agricultural production, its control is still problematic worldwide. Existing management practices of plant-parasitic nematodes (PPNs) predominantly rely on chemical control. However, indiscriminate and long-term use of chemicals has led to considerable environmental pollution and increased nematodes pesticide resistance^5^. Therefore, the development of cost-effective and eco-friendly control strategies is urgently needed. RNA interference (RNAi) is well known to be a feasible tool for gene function analysis, and RNAi-mediated biological control has been reported as a promising strategy for the control of PPNs^6,7^. Disturbing fundamental life processes such as growth, development, and reproduction, by RNAi is considered as a valuable approach for pest control. To date, only few *D. destructor* genes, including the paramyosin gene^8^, venom allergen-like protein gene^9^, alpha-amylase gene^10^ and voltage-gated calcium channel subunit gene^11–13^, have been studied and identified as parasitic or pathogenesis-related genes, which could serve as potential targets for the control of the nematode. Many other putative target genes of *D. destructor* still need to be explored.

Cysteine proteases are well known as crucial hydrolytic enzymes that perform intracellular and extracellular protein degradation, mediating various physiological and pathological processes^14^. Cathepsins belong to the cysteine proteases of papain superfamily and are subdivided into three types based on their sequence diversity and enzymatic activity, including serine cathepsins A and G, aspartic cathepsins D and E, and cysteine cathepsins B, C, F, H, K, L, O, S, V, X and W^15^. Cathepsins are ubiquitous in a variety of organisms, including viruses, plants, invertebrates and vertebrates^16^. In helminth parasites, cathepsins are known to play critical roles in digestion, invasion, migration, immune evasion, growth and development^17–19^. In recent years, cathepsin L-like cysteine protease (CPL) genes have drawn much attention and research efforts in PPNs^20^. PPN CPL gene was first cloned in the cyst nematode *Heterodera glycines*, designated as *hgcp-1*^21^, and it was shown that its downregulation by RNAi resulted in a decrease in female: male ratio^22^. In the root-knot nematode *Meloidogyne incognita*, RNAi-mediated knockdown of the protease gene (*Mi-cpl-1*) depressed the attraction, penetration, and multiplication of the nematode in host plants^23,24^. Silencing of *Bx-cpl* genes using RNAi induced a negative effect on the feeding, reproduction, and pathogenicity of the pine wood nematode *Bursaphelenchus xylophilus*^25^. These results suggested that CPL is a promising target for the control of parasites. However, the CPL genes in *D. destructor* have not yet been studied.

In this study, we functionally characterized a CPL gene *Dd-cpl-1* in *D. destructor*. The *Dd-cpl-1* gene was cloned and its spatiotemporal expression patterns were studied by using reverse transcription-quantitative polymerase chain reaction (RT-qPCR) and in situ hybridization. The physiological functions of *Dd-cpl-1* were analyzed after RNAi-mediated silencing *Dd-cpl-1* by in vitro immersion of the nematode in double-strand RNA *Dd-cpl-1* (ds*Dd-cpl-1*) solution. Our findings suggest that *Dd-cpl-1* plays essential roles in ontogenesis, including embryonic development, J3-J4 transition and female fecundity, as well as in infectivity of *D. destructor*, indicating its potential as target gene for the biological control of this important pest.

## Materials and methods

### Nematode culture and sample collection

The inoculation, cultivation and extraction of *D. destructor* Dd01 strain, as well as screening eggs out from the extracted mixed-stage nematodes, were performed as previously described^10^. One-cell stage eggs were collected from the screened eggs by observation under an IX71 inverted microscope (Olympus, Tokyo, Japan). The second-stage juveniles (J2s) were obtained by incubating the screened eggs in sterile water at 25 °C in the dark for 3 days. The synchronized J2s were inoculated in sweet potato roots (approximately 10,000 J2s each) at 25 °C for 60, 108, 192 h as determined by our laboratory, to obtain synchronized J3s, J4s and adults, respectively.

### Sequence analysis of *Dd-cpl-1*

The nucleotide and amino acid sequences of *Dd-cpl-1* were obtained from our previously published genome data of *D. destructor*^26^. The signal peptide and transmembrane helices of Dd-CPL-1 were predicted by using SignalP (http://www.cbs.dtu.dk/services/SignalP/) and DeepTMHMM (https://dtu.biolib.com/DeepTMHMM), respectively. Conserved domains and active sites were determined using the National Center for Biotechnology Information (NCBI) conserved domain database (https://www.ncbi.nlm.nih.gov/Structure/cdd/wrpsb.cgi). N-glycosylation sites were predicted by using NetNGlyc Server (http://www.cbs.dtu.dk/services/NetNGlyc/). Molecular weight (MW) and isoelectric point (pI) were determined by the ProtParam tool (https://web.expasy.org/protparam/). With Dd-CPL-1 amino acid sequence as a query, orthologs in other organisms were searched by BLASTP (https://blast.ncbi.nlm.nih.gov/Blast.cgi?PROGRAM=blastp&PAGE_TYPE=BlastSearch&LINK_LOC=blasthome) on the NCBI website. Multiple sequence alignments of Dd-CPL-1 and its orthologs were performed using ClustalW (https://www.genome.jp/tools-bin/clustalw), and a phylogenetic tree was constructed using MEGA11.0 software (https://www.megasoftware.net/) with the maximum likelihood (ML) method for 1000 bootstraps. The predicted three-dimensional structure of Dd-CPL-1 was modelled using the online SWISS-MODEL server (https://swissmodel.expasy.org/), and further analyzed via the PyMOL program (https://pymol.org/2/).

### Developmental stage expression patterns of *Dd-cpl-1*

The expression levels of *Dd-cpl-1* in different developmental stages were determined by RT-qPCR . All samples including approximately 10,000 eggs, 5000 J2s, 500 J3s, 500 J4s, 200 female adults and 200 male adults were collected. Total RNA from each sample was extracted separately using TranZol™ Up Plus RNA Kit (Cat# ER501, TransGen Biotech, Beijing, China) and analyzed with a Nanodrop 2000 spectrometer (Thermo Fisher Scientific, Waltham MA, USA). First-strand complementary DNA (cDNA) was synthesized individually using the PrimeScript™ RT reagent Kit with gDNA Eraser (Cat# RR047Q, Takara, Dalian, China). The *alpha-tubulin* (*tba-1*) and *actin* (*act-1*) genes from *D. destructor* were used as internal reference genes^10^. The primers are listed in Table 1. The *tba-1* and *act-1* genes have been widely used as reference genes for RT-qPCR in various plant-parasitic nematodes since they are expressed constitutively in most cells and tissues. RT-qPCR was performed using the SYBR® Green Realtime PCR Master Mix (Cat# QPK-201 T, Toyobo, Osaka, Japan) and an ABI QuantStudio™ 5 Real-Time PCR System (Thermo Fisher Scientific, Waltham MA, USA). The relative expression level of Dd-CPL-1 was calculated by the 2^−ΔΔCt^ method^27^. These experiments were conducted with three independent replicates.

**Table 1:** Primers used for this study.

Primer Name	Sequence (5′ to 3′)	Annotation
*Ddcpl*-qF	GATTGTTCTGCTGCCTAC	RT-qPCR
*Ddcpl*-qR	AGTGTCATCTGCTCCTAC	
*tba-*qF	ACATTCTTCAGTGAGACGCA	RT-qPCR
*tba-*qR	ACCTTGGAGACCGTGACATT	
*act-*qF	AGGTTGCCGCTTTGGTCG	RT-qPCR
*act-*qR	CTTCTGTCCCATTCCGACCA	
*Ddcpl*-cF	ATGTGGAAGCGAATGCTG	cDNA cloning
*Ddcpl*-cR	TTAGACGAGCGGATAGCT	
ISH-S	GGTACTCGGAGAACGGCA	In situ hybridization
ISH-A	GTGACCTCTGCAAGCAG	
ds*Ddcpl*-F	TAATACGACTCACTATAGGG GTGACCTCTGCAAGCAG	dsRNA synthesis
ds*Ddcpl*-R	TAATACGACTCACTATAGGG GGTACTCGGAGAACGGCA	
ds*GFP*-F	TAATACGACTCACTATAGGG GAGTGCCATGCCCGAAGGT	dsRNA synthesis
ds*GFP*-R	TAATACGACTCACTATAGGG GGTCTGCTAGTTGAACGCT	

### Complementary DNA cloning of *Dd-cpl-1*

The total RNA from mixed-stage nematodes was extracted, and the cDNA was synthesized as described above. The encoding sequence of *Dd-cpl-1* was cloned from cDNA using *Ex Taq*® DNA Polymerase (Cat# RR001Q, Takara, Dalian, China) with a pair of specific primers: *Ddcpl-*cF and *Ddcpl-*cR (Table 1). The amplified products were purified and ligated to the pMD^TM^19-T vector (Cat# 6013, Takara, Dalian, China) for DNA sequencing.

### In situ hybridization of *Dd-cpl-1*

To determine the localization of *Dd-cpl-1*, in situ hybridization was performed in different developmental stages of *D. destructor*. Specific sense (ISH-S) and antisense (ISH-A) primers (Table 1) were designed to amplify a 343 bp fragment based on the cloned PCR product of *Dd-cpl-1*. The purified PCR fragment was used as template to synthesize the digoxigenin (DIG)-labeled sense and antisense RNA probes using a DIG-labelling Kit (Cat# DDLK-010, MyLab, Beijing, China). In situ hybridization was performed as previously described^28^ following the manufacturer’s protocol of the DIG hybridization detection Kit (Cat# DIGD-120, MyLab, Beijing, China). Final observations and photographs of nematode specimens were made using an 80i optical microscope (Nikon, Tokyo, Japan).

### Double-stranded RNA (dsRNA) synthesis, RNA interference and phenotype analysis

The dsRNA fragments for *Dd-cpl-1* and the green fluorescence protein (*GFP*) gene were prepared in vitro using T7 RNAi Transcription Kit (Cat# TR102-01, Vazyme, Nanjing, China) and the primers listed in Table 1. The quality and concentration of dsRNA were estimated by gel electrophoresis and Nanodrop 2000 (Thermo Fisher Scientific, Waltham MA, USA).

To investigate the effect of *Dd-cpl-1* silencing on embryonic development, RNAi was performed on 1-cell stage eggs. Approximately 2000 eggs were washed with DEPC water, from which 30 1-cell stage eggs were picked and placed in 96-well plates (Scoperta Life Sciences, Wayne, USA) (one egg per well), followed by adding 50 μg dsRNA in 50 μL. The plates were maintained at 25 °C in a dark incubator with 70% relative humidity. Development of these treated eggs was observed and recorded every 6 h under an IX71 inverted microscope (Olympus, Tokyo, Japan) till hatching. The hatching rates (number of hatched eggs/total number of eggs) were calculated. The rest of the washed eggs were soaked into dsRNA (final concentration 1 μg/μL) for 24 h, and used for qPCR analysis to check the RNAi efficiency.

The effect of RNAi during post-embryonic development was also analyzed by soaking juvenile nematodes into dsRNA. Several thousands of synchronized J2s, J3s, J4s respectively, were washed with DEPC water and soaked into dsRNA (final concentration 1 μg/μL) soaking solution^10^ (50 mM octopamine hydrochloride, 3 mM spermidine and 0.01% TritonX-100) (Sigma-Aldrich, St Louis, MO, USA) for 24 h in a dark rotator (10 rpm) at 25 °C. Then, these dsRNA-treated nematodes at the different juvenile stages were separately inoculated onto small slices (1 × 1 × 0.5 cm) of sweet potato roots (approximately 200 individuals each) and cultured at 25 °C in the dark incubator with 70% relative humidity. The proportion and body size (length and width) of nematodes from one developmental stage to the next stage were observed and recorded at the specific time according to the developmental timeline of untreated nematodes (J2-J3-J4-adults: 60–48–84 h). The rest of the soaked juveniles were retained for checking the RNAi efficiency by qPCR analysis.

To analyze the potential effects of *Dd-cpl-1* RNAi on the nematode reproduction, the quantity and quality of eggs laid by dsRNA-treated females were analyzed. Male and female J4s were collected and cultured separately onto two different sweet potato root slices for 84 h to get virgin adult nematodes. Approximately 200 virgin female adults were collected and incubated in the dsRNA (final concentration 1 μg/μL) soaking solution for 24 h. The mating assay was performed as described^29^ with some modifications. Briefly, after soaking, 30 of the soaked female adults were transferred into 96-well plates (Scoperta Life Sciences, Wayne, USA) (one female per well), followed by adding 100 μL sterile water. Non-RNAi treated virgin male adults were added into these plates (one male per well), and the plates were stood upright to allow that males and females could easily contact each other inducing a successful mating. The number and hatching rates of laid eggs were recorded every 24 h under an IX71 inverted microscope (Olympus, Tokyo, Japan). The rest of the soaked female adults were retained for qPCR analysis to check the RNAi efficiency.

To detect the effect of *Dd-cpl-1* RNAi silencing on the infectivity of *D. destructor*, the number of colonizing nematodes and infection area of dsRNA-treated nematodes in sweet potatoes were analyzed. Mixed-stage nematodes treated with dsRNA soaking solution as mentioned above were inoculated in sweet potato roots (approximately 500 nematodes each). The inoculated roots were then maintained at 25 °C in a dark incubator with 70% relative humidity. After 25 days, the nematodes were collected from the inoculated roots as described before, and counted. The infection area was photographed using a Canon EOS RP digital camera (Canon, Tokyo, Japan) and measured via ImageJ (https://imagej.net/software/imagej/). All pictures from each biological replicate were taken under the same conditions and the infection area was measured by using the ImageJ software. The results were normalized as the percentage value relative to the ds*GFP* control.

The same quality and quantity of nematodes treated with ds*GFP* were used as control. All experiments were performed at least in triplicate with three biological replicates.

### Data analysis

For statistical analyses, the relative expression levels among the developmental stages were analyzed with a one-way analysis of variance (ANOVA) followed by Tukey’s HSD test, using SPSS 22.0 (IBM, Chicago, IL, USA). Different letters indicate significant differences in corresponding expression levels (*P* < 0.05). The significant differences of two groups were determined with unpaired Student’s *t*-test (ns, not significant, *P < 0.05; **P < 0.01; ***P < 0.001; ****P < 0.0001) using GraphPad Prism 8 (Graphpad Software, La Jolla, CA, USA).

## Results

### Sequence and phylogenetic analysis of *Dd-cpl-1*

**Figure 1:**
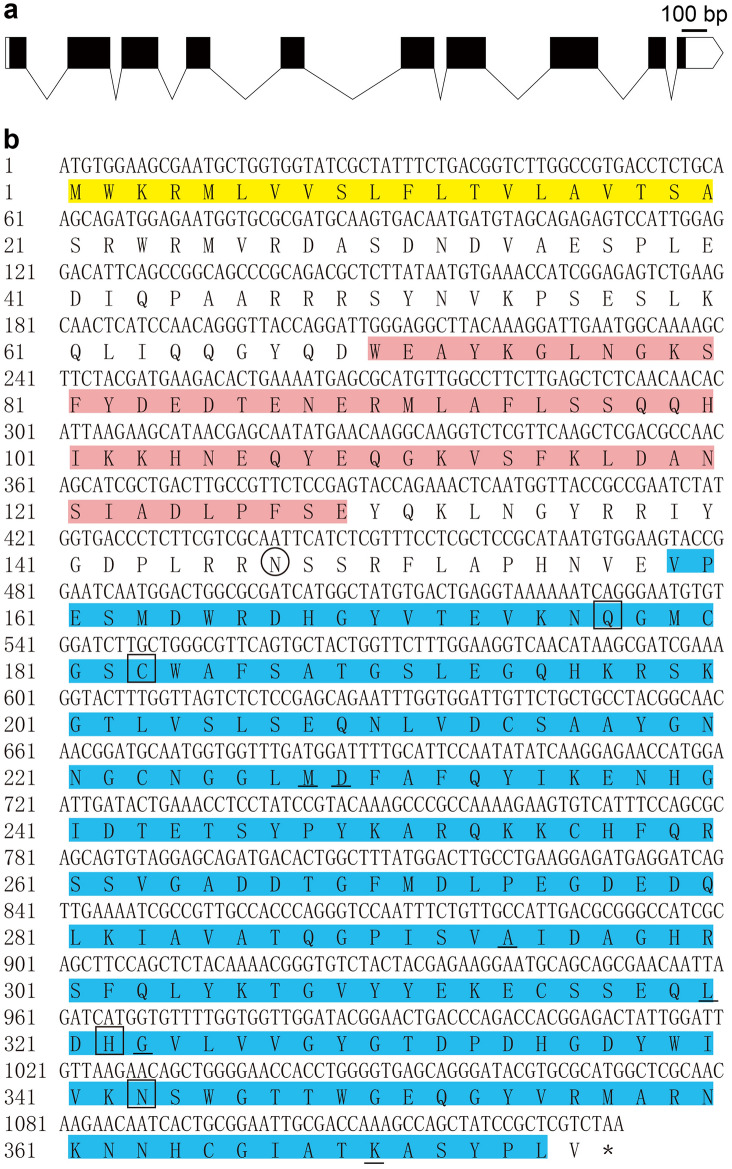
Sequence analysis of *Dd-cpl-1*. (**a**) Gene structure of *Dd-cpl-1*. Relative positions and respective sizes of exons are indicated as boxes and introns as lines; coding sequences are indicated as black boxes; untranslated regions are indicated as white boxes. (**b**) Nucleotide and amino acid sequences of *Dd-cpl-1*. The predicted signal peptide is highlighted in yellow. The I29 inhibitor domain is highlighted in pink. The peptidase C1 domain is highlighted in blue. The potential *N*-glycosylation site is circled. S2 subsites are boxed. Active site residues are underlined.

The *Dd-cpl-1* gene sequence was obtained based on our previously published genome data of *D. destructor*^26^. The nucleotide sequence of *Dd-cpl-1* (Gene ID in WormBase ParaSite Database: *Dd_06582*) spans 2841 bp on *D. destructor* genome. This gene has only one transcript composing of ten exons and nine introns (Fig. 1a). *Dd-cpl-1* mRNA encompasses 16 bp of the 5′ untranslated region (UTR) and 149 bp of the 3′ UTR, and has an ORF of 1131 bp encoding a protein of 376 amino acids (aa) with a 20 aa signal peptide. Dd-CPL-1 N-terminus contains a I29 inhibitor domain (I29I), which may act as a propeptide and prevent access of the substrate to the active site. A peptidase C1 domain (PC1) as a mature peptide was identified in the C-terminus, and six S2 subsites defined by residues Leu227, Met228, Ala294, Leu320, Gly323 and Lys370 were found within this domain. A potential N-glycosylation site located at Asn147 and the four active site residues Gln177, Cys183, His322 and Asn343 were also identified (Fig. 1b). No transmembrane region was predicted in Dd-CPL-1. The estimated MW and theoretical pI of the protein were 42.51 kDa and 6.14, respectively.

**Figure 2:**
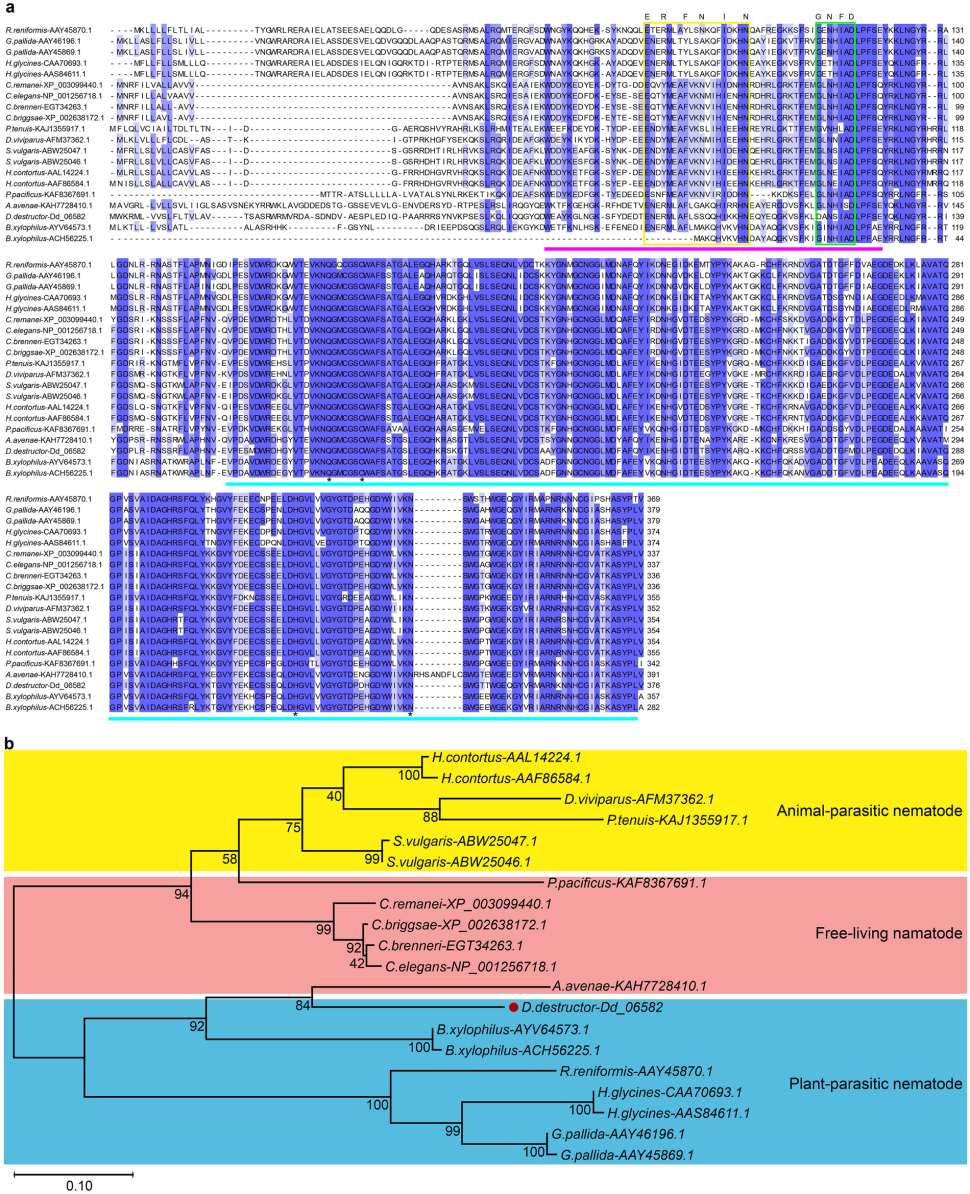
Alignment and phylogenetic analysis of *Dd-cpl-1* with 19 orthologs. The Gene ID of *Dd-cpl-1* (*Dd_06582*) in WormBase ParaSite Database and GenBank accession numbers of the 19 orthologs in the NCBI database followed each species. (**a**) Multiple sequence alignment of *Dd-cpl-1* with the 19 orthologs. The ERFNIN and GNFD motifs of CPLs are enclosed by the yellow and green boxes, respectively. The I29 inhibitor domain is marked by purple rectangle. The peptidase C1 domain is marked by cyan rectangle. Conserved catalytic residues are indicated by asterisks. (**b**) A phylogenetic tree of the 20 CPLs was constructed with the maximum likelihood method. The numbers on the branches correspond to the percentage of 1000 bootstrap value that support each branch. Nematode species with different living strategies are highlighted using different color backgrounds.

The sequences of nineteen orthologs from different nematodes, including *Aphelenchus avenae* (KAH7728410.1), *Bursaphelenchus xylophilus* (ACH56225.1, AYV64573.1), *Globodera pallida* (AAY45869.1, AAY46196.1), *Heterodera glycines* (CAA70693.1, AAS84611.1), *Meloidogyne incognita* (CAD89795.1), *Rotylenchulus reniformis* (AAY45870.1), *Haemonchus contortus* (AAL14224.1, AAF86584.1), *Dictyocaulus viviparus* (AFM37362.1), *Parelaphostrongylus tenuis* (KAJ1355917.1), *Caenorhabditis brenneri* (EGT34263.1), *Caenorhabditis elegans* (NP_001256718.1), *Caenorhabditis briggsae* (XP_002638172.1), *Caenorhabditis remanei* (XP_003099440.1) and *Pristionchus pacificus* (KAF8367691.1) were obtained from NCBI, with their corresponding GenBank accession numbers listed inside the brackets. Multiple sequence alignment analysis showed that Dd-CPL-1 shared considerable evolutionary conservation with other nematode CPLs, showing an overall identity of 56.63–75.34%. Moreover, a conserved Gln residue, and three conserved residues (Cys, His, and Asn) that might form a catalytic triad to catalyze the hydrolysis of peptide bonds in the substrates, were identified in all CPLs. Also, the relatively conserved ERFNIN and GNFD motifs, and the highly conserved I29I and PC1 were detected in all CPLs (Fig. 2a). Phylogenetic tree analysis of the same 20 protein sequences indicated that CPLs were widely distributed in a variety of nematode species with different living strategies (free-living, plant-parasitic and animal-parasitic) and were clustered according to their living strategies. Furthermore, CPLs from nematodes (*A. avenae*, *D. destruvtor* and *B. xylophilus*) that can feed on fungi were clustered in the same branch (Fig. 2b).

### Homology modeling of Dd-CPL-1

**Figure 3:**
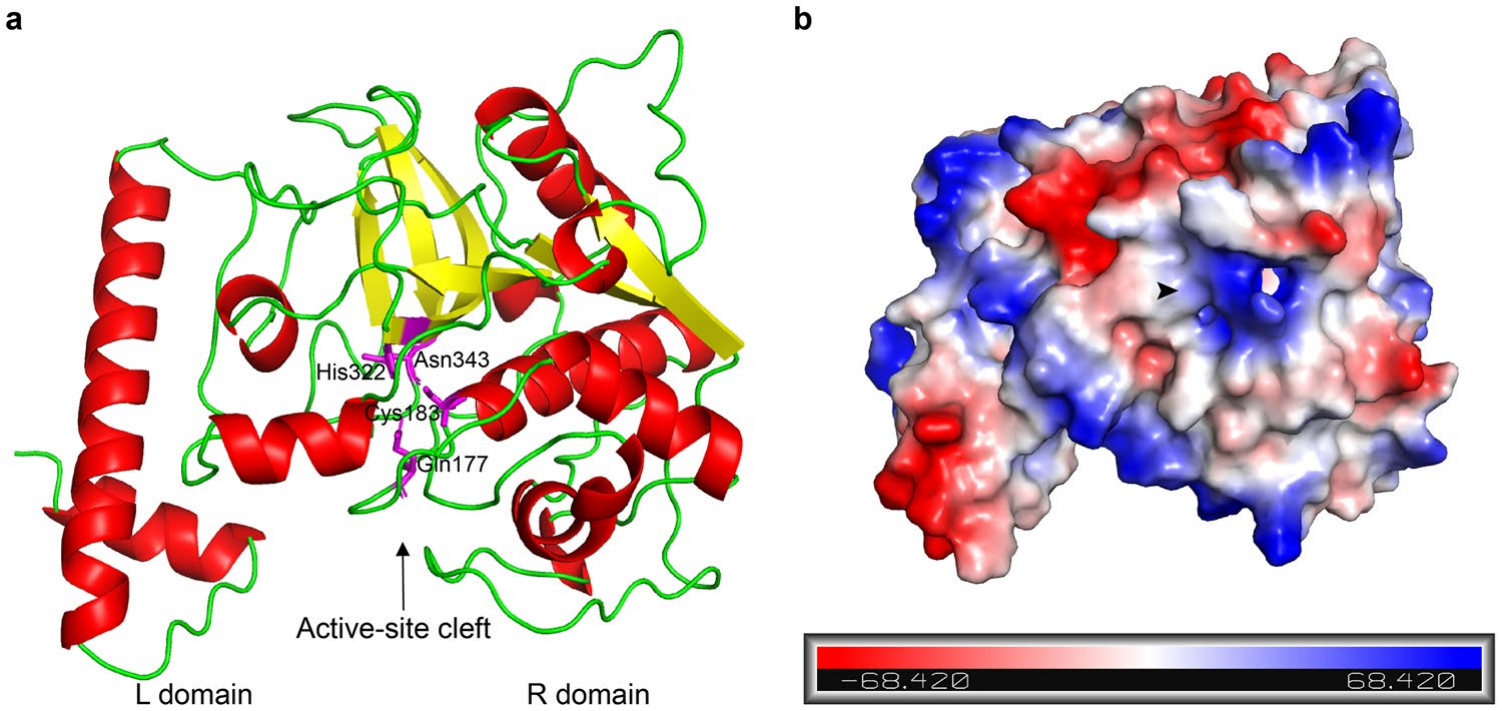
Molecular modeling of Dd-CPL-1. (**a**) The predicted three-dimensional structure of Dd-CPL-1 composed of the L and R domains was made by SWISS-MODEL with human cathepsin L (PDB: 6jd8.1.A) as a template. The L and R domains are divided by an active-site cleft (indicated by the arrow). Four active site residues within the active-site cleft are highlighted in purple. (**b**) The electrostatic potential surface of Dd-CPL-1. Positively- and negatively-charged regions are indicated by blue and red color, respectively; the higher the charge quantity, the darker the color. Neutral regions are indicated by white color. A predicted hydrophobic pocket is indicated by the arrowhead.

The three-dimensional structure of Dd-CPL-1 was predicted by using the online SWISS-MODEL server with the crystal structure of human cathepsin L (PDB: 6jd8.1.A) as template. The fold of Dd-CPL-1 consisted of two domains, the L and R domains, divided by the active-site cleft, with the L domain dominated by alpha-helices and the R domain composed of alpha-helices and beta-strands (Fig. 3a). Molecular surface modelling was calculated using the PyMOL program based on the structure of Dd-CPL-1, showing the presence of charged regions and neutral regions. A hydrophobic pocket that can accommodate residues of the substrate was predicted to be located in the S2 subsites (Fig. 3b).

### Expression pattern and tissue localization of *Dd-cpl-1*

**Figure 4:**
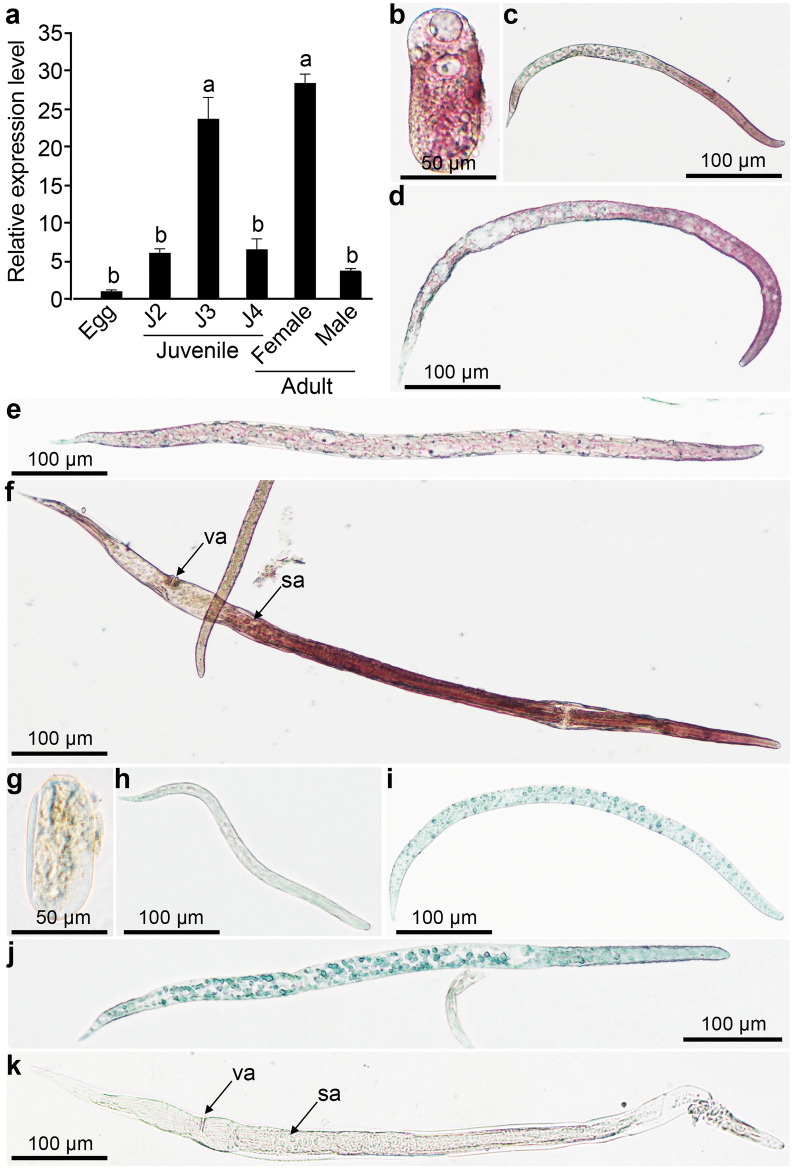
The expression pattern and tissue localization of *Dd-cpl-1* in *D. destructor*. (**a**) Temporal expression patterns of *Dd-cpl-1* at different development stages. Data shown are means ± standard errors. Significant differences (*P* < 0.05) are indicated by different letters. (**b**-**k**) Localization of *Dd-cpl-1* mRNA in *D. destructor* using in situ hybridization with the digoxigenin-labelled specific probe in the egg (**b**), second-stage juvenile (**c**), third-stage juvenile (**d**), fourth-stage juvenile (**e**) and female adult (**f**). No signal in eggs and worms were hybridized with the control sense probe (**g**-**k**). va, vulva; sa, spermatheca.

*Dd-cpl-1* was expressed at all developmental stages in *D. destructor*. Relatively higher expression levels were detected in J3s and female adults (Fig. 4a). Using the expression level in eggs as reference, we found that *Dd-cpl-1* was upregulated 6.03- and 23.65-fold in J2, J3, respectively. Then it went down to 6.52-fold in J4 stage. Finally, in adult stage expression level of *Dd-cpl-1* remained down (3.71-fold) in male adults, while it showed upregulation (up to 28.34-fold) in female adults.

In situ hybridization showed that *Dd-cpl-1* was located in the bulk of eggs, juveniles and female adults (Fig. 4b–f). The digoxigenin-labelled specific probe primarily stained the esophagi and intestines in J2s (Fig. 4c), J3s (Fig. 4d), J4s (Fig. 4e) and female adults (Fig. 4f). In addition, a clear hybridization signal was also detected in the ovaries and spermathecae of female adults (Fig. 4f). No signal was observed in the control worms and eggs that were incubated with the digoxigenin-labelled sense probe (Fig. 4g–k).

### Effect of *Dd-cpl-1* silencing on *D. destructor* embryogenesis

**Figure 5:**
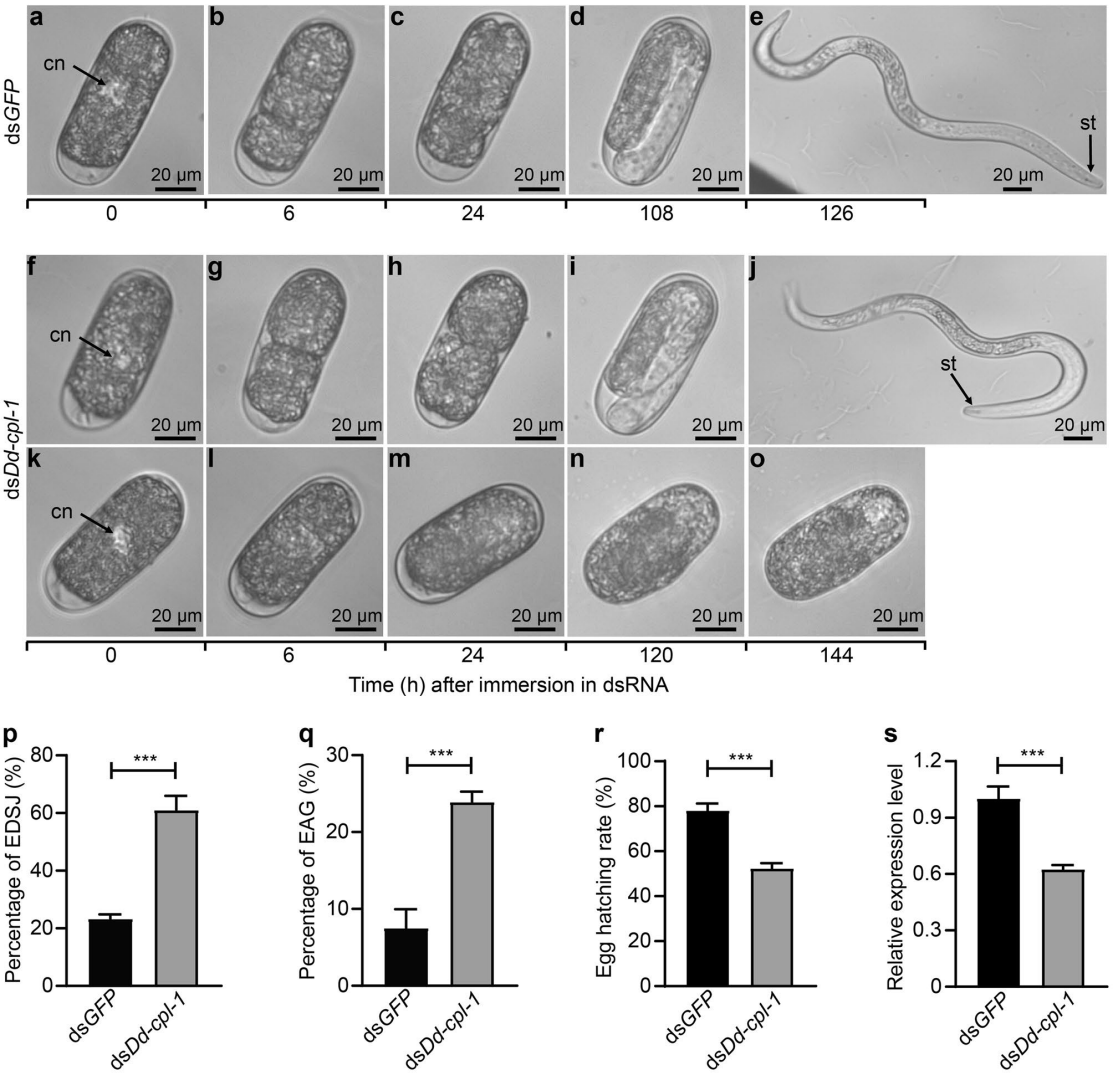
Effects of *Dd-cpl-1* silencing on *D. destructor* embryos. Nematode eggs soaked with ds*GFP* (**a**-**e**) or ds*Dd-cpl-1* (**f**-**o**); 1-cell stage (**a**, **f**, **k**); 3-cell stage (**b**); multi-cell stage (**c**); first-stage juvenile (**d**, **i**); second-stage juvenile (**e**, **j**); early 3-cell stage (**g**); 4-cell stage (**h**); late 1-cell stage (**l**); early 2-cell stage (**m**); blastula stage (**n**); gastrula stage (**o**). cn, cell nucleus; st, stylet. Percentages of eggs that develop slowly to J2 (EDSJ) (**p**) and eggs that arrest at gastrula stage (EAG) (**q**) after treatments with ds*Dd-cpl-1* or ds*GFP*. Bar graphs show the EDSJ percentage (number of EDSJ/total number of J2s) and EAG percentage (number of EAG / total number of input eggs). (**r**) Egg hatching rates after treatments with ds*Dd-cpl-1* or ds*GFP*. EDSJ, EAG and J2s were counted after egg immersion in dsRNA solution for 144 h. (**s**) The expression levels of *Dd-cpl-1* detected in eggs after treatments with ds*Dd-cpl-1* or ds*GFP*. Data shown are means ± standard errors from three biological replicates. The *P*-value was calculated by unpaired Student’s *t*-test (***P < 0.001).

RNAi analysis showed that the control eggs treated with ds*GFP* completed embryonic development from 1-cell egg to J1 and J2 stages in108 and 126 h, respectively (Fig. 5a–e), whereas the ds*Dd-cpl-1*-treated eggs underwent abnormal development. First, approximately 60% of the ds*Dd-cpl-1*-treated eggs that were able to developed into J2 stage showed a delayed development phenotype (Fig. 5f–j), requiring up to 120 and 144 h to reach J1 (Fig. 5i) and J2 (Fig. 5j), respectively, which was significantly delayed compared with the control eggs (*P* = 0.0002; Fig. 5p). Second, approximately 20% of the ds*Dd-cpl-1*-treated eggs displayed prolonged development during early embryogenesis, arrestment at gastrula stage and could not undergo any further morphogenesis (Fig. 5k–o), which was markedly higher than that in the control group (*P* = 0.0005; Fig. 5q). Finally, the hatching rate of ds*Dd-cpl-1*-treated eggs was significantly lower than that in the ds*GFP* control group by 25.71% (*P* = 0.0003; Fig. 5r). The analysis of *Dd-cpl-1* transcript by qPCR results showed that the expression level of *Dd-cpl-1* was significantly suppressed after RNAi with ds*Dd-cpl-1* compared with ds*GFP* (*P* = 0.0007; Fig. 5s).

### Effect of *Dd-cpl-1* silencing on juvenile development

**Figure 6:**
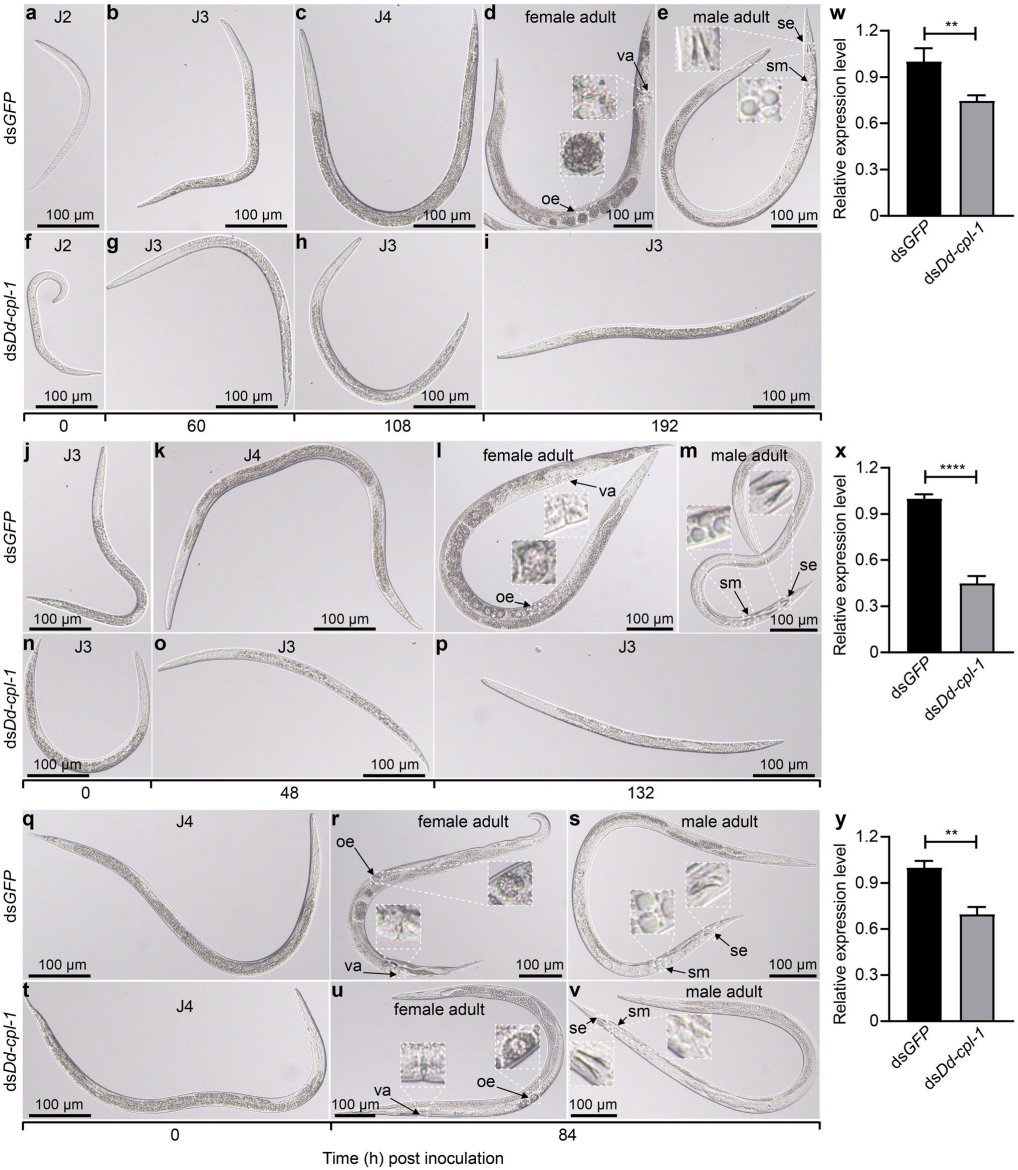
Effects of *Dd-cpl-1* silencing on *D. destructor* juveniles. The development of juveniles after treatments with ds*GFP* (**a**-**e**, **j**-**m**, **q**-**s**) or ds*Dd-cpl-1* (**f**-**i**, **n**-**p**, **t**-**v**). The inserts show enlarged images of vulva (va), oocyte (oe), spicule (se), and sperm (sm). The expression levels of *Dd-cpl-1* detected in J2s (**w**), J3s (**x**), J4s (**y**) after treatments with ds*Dd-cpl-1* or ds*GFP*. Data shown are means ± standard errors from three biological replicates. The *P*-value was calculated by unpaired Student’s *t*-test (**P < 0.01; ****P < 0.0001).

RNAi-mediated silencing of *Dd-cpl-1* at juvenile stages J2 and J3 inhibited the development of J3s into adults. At 60 h post-inoculation (hpi), the vast majority of ds*Dd-cpl-1*-treated J2s entered J3 (Fig. 6f, g), which was comparable to the ds*GFP* control group (Fig. 6a, b). However, at 108 and 192 hpi, most of these control J2s reached J4 (Fig. 6c) and adult (Fig. 6d, e) stages, respectively, whereas the ds*Dd-cpl-1*-treated J2s still stayed at J3 (Fig. 6h, i and Fig. S1a). Similarly, at 48 and 132 hpi, the control J3s reached J4 (Fig. 6j, k) and adult stages (Fig. 6l, m), respectively. In contrast, few of the ds*Dd-cpl-1* interfered J3s entered these stages (Fig. 6n–p and Fig. S1b). Finally, when RNAi was done in J4s, there was no significant difference in growth rates between the control group and the ds*Dd-cpl-1* group. At 84 hpi, almost all J4s treated with ds*Dd-cpl-1* (Fig. 6t–v), or with the control ds*GFP* (Fig. 6q–s and Fig. S1c) developed into adults and no remarkable changes were detected in body length and width for female and male adults (data not shown). The qPCR results revealed that after 24 h of soaking J2, J3, J4 nematodes in ds*Dd-cpl-1*, the expression levels of *Dd-cpl-1* were all significantly reduced (Fig. 6w–y).

### Effect of *Dd-cpl-1* silencing on female fecundity and infectivity

**Figure 7:**
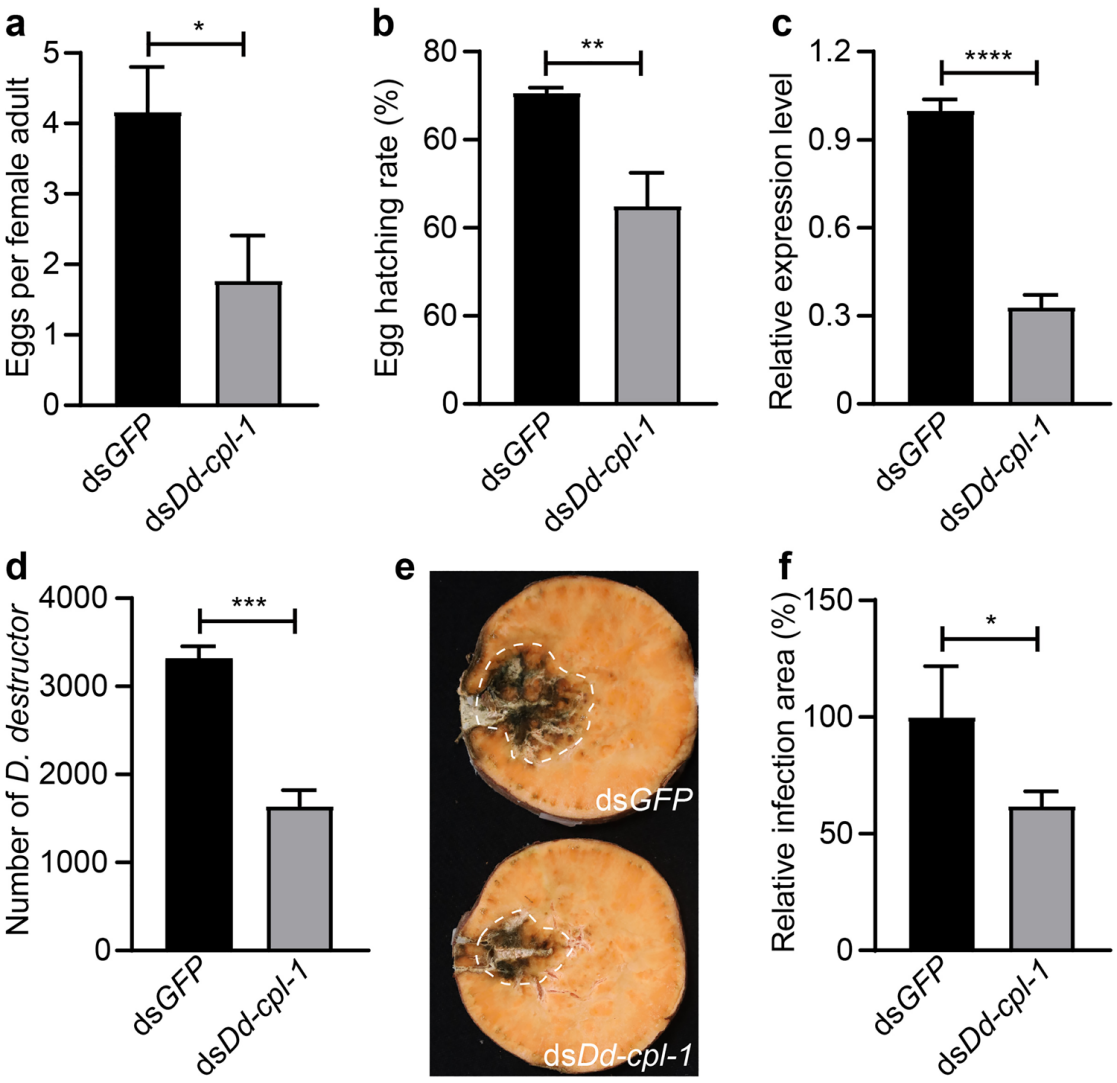
Effects of *Dd-cpl-1* silencing on fecundity and infectivity of *D. destructor*. (**a**) Female fecundity analysis after the treatments with ds*Dd-cpl-1* or ds*GFP*. Eggs and J2s were counted after nematode pairing for eight days. Egg production means the sum of the number of eggs and J2s. (**b**) Hatching rates of eggs laid by female adults treated with ds*Dd-cpl-1* or ds*GFP*. Eight days after nematode pairing, the percentage of J2s was determined. (**c**) The expression levels of *Dd-cpl-1* detected in female adults after treatments with ds*Dd-cpl-1* or ds*GFP*. (**d**) The colonization numbers of *D. destructor* in the sweet potato storage roots infected with ds*Dd-cpl-1*- or ds*GFP*-treated mixed-stage nematodes at 25 d post-infection. (**e**) The infection areas highlighted with a dashed border. (**f**) The infection areas indicated as the percentage value relative to the ds*GFP* control. Infection area was calculated by using ImageJ. Data shown are means ± standard errors from three biological replicates; each replicate contained 30 female adults for fecundity, and contained five technic replicates for infectivity. *P*-values were calculated by unpaired Student’s *t*-test (*P < 0.05; **P < 0.01; ***P < 0.001, ****P < 0.0001).

Compared with the ds*GFP* control group, the average number of eggs laid by ds*Dd-cpl-1*-treated female adults was significantly decreased by 57.43% (*P* = 0.01) after 8 days (Fig. 7a). Furthermore, the hatching rate of the eggs that were laid by ds*Dd-cpl-1*-treated females was 25.68% (*P* = 0.0041) lower than the control group (Fig. 7b). The majority of eggs (approximately 60%) in the ds*Dd-cpl-1* group could not normally hatch, and eventually died. As expected, soaking female adults in ds*Dd-cpl* for 24 h significantly diminished the expression of *Dd-cpl-1* (Fig. 7c).

Our findings suggest that *Dd-cpl-1* plays important roles in embryogenesis, juvenile development and female fecundity in *D. destructor*. We used mixed-stage nematodes to test the effect of *Dd-cpl-1* silencing on infectivity. The number of nematodes colonizing the sweet potato storage roots after treatment with ds*Dd-cpl-1* was significantly lower than that in the ds*GFP* control group by 50.51% (*P* = 0.0002; Fig. 7d). Meanwhile, the infection area of the nematodes treated with ds*Dd-cpl-1* was noticeably decreased compared to the control group (*P* = 0.0441; Fig. 7e, f). These findings indicate that *Dd-cpl-1* plays a role in *D. destructor* infectivity.

## Discussion

Cathepsin L-like cysteine protease (CPL) is one of the most important cellular proteases and plays key roles in nematodes and many other animal parasites^18,30^. CPL genes have been identified and cloned from a few PPN species where all functional investigations were conducted using RNAi^22–25,31^. However, the biological functions of CPL genes in the potato rot nematode *D. destructor* have yet to be explored. Herein, a CPL gene, *Dd-cpl-1*, from *D. destructor* was functionally characterized.

Amino acid sequence and multiple sequence alignment analyses revealed that Dd-CPL-1 exhibits domain architecture with the presence of a signal peptide followed by I29I and PC1 domains, and four conserved catalytic residues of Gln, Cys, His, and Asn, as found in other CPLs from free-living nematode^32^ and insects^33^. ERFNIN and GNFD motifs located in the I29I domain are relatively conserved and they are the main signatures of CPLs^34,35^. Phylogenetic analysis indicated that Dd-CPL-1 has the closest evolutionary phylogenetic relationship with the ortholog from the free-living fungivorous nematode *A. avenae* sharing 75.34% amino acid sequence identity. Molecular modelling showed that the structure of Dd-CPL-1 is composed of two globular domains divided by an active-site cleft, which is quite similar to that of other nematodes, such as *B. xylophilus*^36^, *Angiostrongylus cantonensis*^37^ and *Trichinella spiralis*^38^. These results suggest that the CPL genes are conserved across the nematode phylum and Dd-CPL-1 may share similar functions with its orthologs in other nematode species.

Some previous studies have confirmed that CPL genes are essential for embryonic development in nematodes. For example, silencing of *Bm-cpl* genes (*Bm-cpl-1*, *Bm-cpl-4* and *Bm-cpl-5*) in filarial nematode *Brugia malayi* resulted in malformed intrauterine embryos and abnormal embryonic viability^39^. *C. elegans cpl-1* mutant embryos showed decreased cell division rate, arrest of morphogenesis and eventual death, and it was shown that Ce-CPL-1 was involved in the degradation of yolk^32,40^, which provides the major nutrients for developing embryos. Transgenic expression of the *H. contortus cpl-1* or *S. vulgaris cpl-1* genes rescued the embryonic lethal phenotype of the *C. elegans cpl-1* mutant, supporting their function in embryonic development^41,42^. Here, we show that *Dd-cpl-1* RNAi embryos of *D. destructor* shared similar phenotypes with the *C. elegans cpl-1* mutant, such as the dramatically slow cell division, abortive morphogenesis and embryonic lethal phenotype. These results may be explained by the potential participation of Dd-CPL-1 in yolk processing in *D. destructor*, which resembles the function of Ce-CPL-1 in *C. elegans*^40^. This hypothesis requires to be demonstrated in the future.

The expression level of *Dd-cpl-1* in J3s was significantly higher than that in J2s, and J4s, which indicates that CPL may be one of the enzymes required for a specific, tightly regulated process during J3 development. Cell growth and proliferation depend on the nutritional status. A programmed arrest of development, diapause has been found at some stages of ontogenesis in many animals, and it has been shown that the diapause state may be controlled by the nutritional status^43^. The mechanistic target of rapamycin (mTOR) pathway can sense and integrate the nutritional status to regulate eukaryotic cell growth^44^. In *C. elegans*, TOR-deficient worms appeared to be defective in digestion or nutrient absorption, and arrested at third-stage larvae (L3)^45^. Other nutrient sensing pathways have also been identified in *C. elegans*, such as the Insulin/insulin-like growth factor (IGF-1) signaling (IIS) pathway^46^. IIS pathway mutants (*Ce-daf-2* and *Ce-age-1*) arrested development in the dauer stage, an alternative third larval stage^47^. It is still unclear whether there is a dauer state in parasitic nematodes. CPLs are members of proteolytic enzymes and have been confirmed to be involved in digestion for nutrition in many animals^17,48,49^. Here we found that *Dd-cpl-1* was located in the digestive system and silencing *Dd-cpl-1* expression by RNAi in J3s resulted in developmental arrest in this stage. Thus, it is possible to speculate that Dd-CPL-1 may directly affect *D. destructor* development and growth by acting as a digestive enzyme to supply nutrition, or may play roles in the mTOR or in the IIS pathway through comparison of the phenotype (developmental arrest at third larval stage) after disruption of *Dd-cpl-1*, *CeTOR*, *Ce-daf-2* and *Ce-age-1*^45,50,51^. Additionally, a developmental arrest phenotype in J3s was also observed after RNAi of *Dd-cpl-1* at stage of J2. In contrast, when J4s were treated with *Dd-cpl-1* RNAi these worms were still able to develop into adults comparable to the control nematodes. These findings suggest that diapause and other developmental decisions in *D. destructor* might be determined at J3 stage and during which *Dd-cpl-1* functions are required, which coincide with its relatively high expression in J3s. However, the detailed regulatory mechanisms in developmental decisions remain unknown and need further study.

Theoretically, an efficient gene-silencing might result in more pronounced phenotypic changes, while a lower level of gene-silencing might lead to more subtle or intermediate phenotypes. However, it is important to note that this correlation is not always direct or predictable. The relationship between phenotype levels and silencing efficiency can be influenced by multiple variables, including the complexity of gene interactions, redundancy of biological systems, and influence of environmental factors. Our study showed that even though that the RNAi silencing efficiency of *Dd-cpl-1* in J2s was comparable to that in J4s, their phenotypes were completely different, since the interfered J2s arrest at J3 stage, while the interfered J4s can develop normally into adults. On the other hand, the difference in RNAi silencing efficiency of *Dd-cpl-1* in J2s and J3s is significant, but the interfered J2 and J3 animals showed a similar developmental arrest phenotype at J3 stage.

CPL genes have been reported as being implicated in parasite reproduction. Earlier studies showed that *Schistosoma mansoni* CPL was localized in the reproductive organs and was involved in female fecundity^52,53^. Knocking down *Mi-cpl-1* gene by in vitro RNAi or plant-mediated RNAi resulted in reduced egg number of female *M. incognita*^23,24,31^. Recently, Bai et al.^54^ reported that silencing of *TsCL* gene could significantly decrease newborn larvae production by *T. spiralis* adult females. Similarly, we found that female *D. destructor* with silenced expression of *Dd-cpl-1* were affected in egg production. Besides, our findings also showed that the highest expression level of *Dd-cpl-1* was detected in female adults and the localization of *Dd-cpl-1* was observed in the ovaries and spermathecae of female adults. These results indicate that *Dd-cpl-1* gene also plays an important role in the reproductive capacity of *D. destructor*. Intriguingly, the expression level of *Dd-cpl-1* was remarkably higher in female adults than in male adults, but no noticeable change in female: male ratio after *Dd-cpl-1* silencing was observed (data not shown). It is inferred that *Dd-cpl-1* may perform some other sex-specific functions not yet identified.

Finally, we determined that the infectivity (colonization and infection area) of *D. destructor* was significantly depressed after silencing *Dd-cpl-1* gene. The lower infectivity may be explained since nematodes treated with *Dd-cpl-1* RNAi exhibit defects in egg viability, juvenile development, female fecundity and/or other biological processes. For example, suppression of *Bm-cpl-1* elicited slow moving, incomplete and porous gut, disrupted cuticle and uncompleted molting in *B. malayi*^55^. Overall, our results show that reducing the expression of *Dd-cpl-1* gene has negative pleiotropic effect on *D. destructor* ontogenesis, suggesting that *Dd-cpl-1* gene target for RNAi could be a good strategy for the control of the nematode.

## Conclusion

In summary, we functionally characterized the CPL gene *Dd-cpl-1* in *D. destructor*. Our results showed that *Dd-cpl-1* plays multiple roles throughout the life cycle of this nematode with its essential involvement in embryogenesis, juvenile development and female fecundity. Furthermore, we determined that *Dd-cpl-1* indeed plays a role in nematode infectivity. Our work not only helps to understand the role of *Dd-cpl-1* in *D. destructor* ontogenesis, but also indicates that *Dd-cpl-1* could serve as a good candidate target for future RNAi-based control of *D. destructor*.

### Supplementary Information

Supplementary Information.

## Data Availability

The nucleotide and amino acid sequences of *Dd-cpl-1* are available from our previously published *D. destructor* genome data on the WormBase website (https://parasite.wormbase.org/Ditylenchus_destructor_prjna312427/Info/Index/).
