# Supplementary material for: Silencing Ditylenchus destructor cathepsin L-like cysteine protease has negative pleiotropic effect on nematode ontogenesis
Source: Sci Rep. 2024 May 1;14:10030. doi: 10.1038/s41598-024-60018-5 (PMC11063044; doi:10.1038/s41598-024-60018-5)
Supplement: Supplementary file 1 — Supplementary Information. [file 41598_2024_60018_MOESM1_ESM.pdf]

Supplementary figures

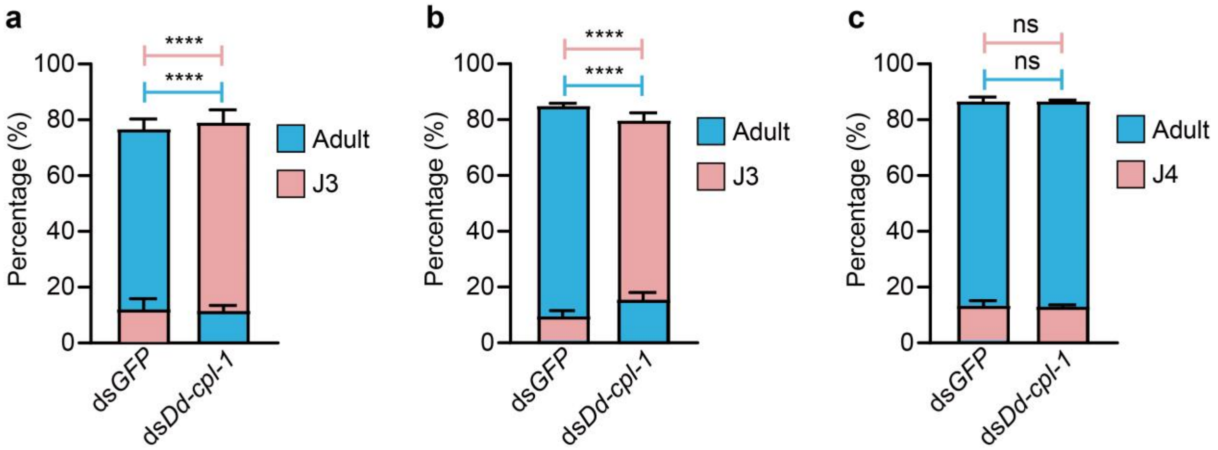

Supplementary Fig. 1. Juvenile development of ds*GFP*- or ds*Dd-cpl-1*-treated *D. destructor*. (**a**) Bar graphs showing the percentage J3s and adults. Approximately 200 dsRNA-treated J2s were inoculated onto small sweet potato slices for 192 h. (**b**) Bar graphs showing the percentage J3s and adults. Approximately 200 dsRNA-treated J3s were inoculated onto small sweet potato slices for 132 h. (**c**) Bar graphs showing the percentage J4s and adults. Approximately 200 dsRNA-treated J4s were inoculated onto small sweet potato slices for 84 h. (**a**)–(**c**) The inoculated nematodes were collected and nematodes at different developmental stages were counted. Data shown are means ± standard errors from three biological replicates; each biological replicate contained five technic replicates. *P*-values were calculated by unpaired Student's *t*-test (ns, not significant, ****, $P < 0.0001$).
